# Construction and validation of prognostic signature for hepatocellular carcinoma basing on hepatitis B virus related specific genes

**DOI:** 10.1186/s13027-022-00470-y

**Published:** 2022-12-06

**Authors:** Lei Wang, Manman Qiu, Lili Wu, Zexing Li, Xinyi Meng, Lu He, Bing Yang

**Affiliations:** 1 Tianjin Second People’s Hospital, Tianjin, 300192 China; 2 Tianjin Institute of Hepatology, Tianjin, 300192 China; 3 College of Life Sciences, Nankai University, Tianjin, 300071 China; 4 Logistics University of People’s Armed Police Force, Tianjin, 300000 China; 5 School of Life Sciences, Tianjin University, Tianjin, 300072 China; 6 Department of Cell Biolopgy, School of Basic Medical Sciences, Tianjin Medical University, Tianjin, 300070 China; 7 Department of Anatomy and Histology, School of Basic Medical Sciences, Tianjin Medical University, Tianjin, 300070 China

**Keywords:** Hepatocellular carcinoma (HCC), Hepatitis B virus (HBV), Prognostic signature, Overall survival

## Abstract

**Background:**

Hepatocellular carcinoma (HCC) is a frequent primary liver cancer, and it is one of the leading cause of cancer-related deaths. Hepatitis B virus (HBV) infection is a crucial risk factor for HCC. Thus, this study aimed to explore the prognostic role of HBV-positive HCC related specific genes in HCC.

**Methods:**

The HCC related data were downloaded from three databases, including The Cancer Genome Atlas (TCGA), International Cancer Genome Consortium (ICGC), and Gene Expression Omnibus (GEO). Univariate Cox regression analysis and LASSO Cox regression analysis were conducted to build the Risk score. Multivariate Cox regression analysis and survival analysis determined the independent prognostic indicators.

**Results:**

After cross analysis of differentially expressed genes (DEGs), we have identified 106 overlapped DEGs, which were probably HBV-positive HCC related specific genes. These 106 DEGs were significantly enriched in 213 GO terms and 8 KEGG pathways. Among that, 11 optimal genes were selected to build a Risk score, and Risk score was an independent prognostic factor for HCC. High risk HCC patients had worse OS. Moreover, five kinds of immune cells were differentially infiltrated between high and low risk HCC patients.

**Conclusion:**

The prognostic signature, based on HMMR, MCM6, TPX2, KIF20A, CCL20, RGS2, NUSAP1, FABP5, FZD6, PBK, and STK39, is conducive to distinguish different prognosis of HCC patients.

**Supplementary Information:**

The online version contains supplementary material available at 10.1186/s13027-022-00470-y.

## Background

Hepatocellular carcinoma (HCC) is a frequent primary liver cancer, and it has ranked the second leading cause of various cancers’ mortality up to 2020 [1]. The incidence of HCC has been reported to increase during the past decades, and over 90,000 new cases of HCC are estimated in 2020 [2]. Many risk factors for developing HCC have been widely investigated, such as non-alcoholic steatohepatitis, chronic hepatitis B or C virus infections, and progressive fibrotic liver diseases [3–5]. Only a small part of HCC patients (about 20%) could be diagnosed at an early stage, and these patients are more probably eligible for surgical therapies or radiofrequency ablation [6]. As for those undetectable HCC patients, oral tyrosine-kinase inhibitor (TKI) sorafenib has been the first-line treatments with survival benefit and enough safety [7, 8]. However, due to high metastasis and recurrence rate, the long term prognosis of HCC patients is still poor, and the 3-year and 5-year overall survival (OS) rate is less than 20% [9, 10]. Accordingly, increasing diagnostic or prognostic biomarkers/ signatures are expected to improve the outcome of HCC patients directly or indirectly, such as hypoxia-related prognostic signature [11], immune-related signature [12], and so on.

Hepatitis B virus (HBV) and hepatitis C virus (HCV) infections are dominating risks causing HCC, among which HBV is a heavier healthy burden in China [13–15]. HBV, as a small hepatotropic DNA virus, could result in acute or chronic liver diseases, thereby leading to hepatic damage, fibrosis and liver cancer [16, 17]. Before the transformation from HBV infection to HCC, there is a long-time interaction between HBV and host hepatocytes, comprising HBV DNA integration, aberrant regulatory protein expression, and epigenetic dysregulation [18]. Moreover, in most adult patients, HBV infection could lead to a rapid immune response and acute self-limited infection [19]. Currently, increasing HBV based biomarkers or signatures have suggested their favorable potential regarding the prognosis or diagnosis of HCC patients. PYCR2 (pyrroline-5-carboxylate reductase 2) and ADH1A (alcohol dehydrogenase 1 A (class I), alpha polypeptide) are recently identified as prognostic biomarkers in HBV-related HCC, involving metabolic reprogramming [20]. Moreover, Yan et al. have built an OS predictive signature based on 4 genes in HCC [21]. A two-m6A-regulator based prognostic signature has been reported in HBV-related HCC [22]. Whereas, as far as we know, few prognostic signatures based on HBV related specific genes in HCC have been reported.

In this study, we mainly aimed to explore the prognostic value of HBV-positive related genes in HCC patients. Integrating HCC data from The Cancer Genome Atlas (TCGA), International Cancer Genome Consortium (ICGC), and Gene Expression Omnibus (GEO) databases, multiple bioinformatic analyses were conducted in order to construct a reliable prognostic signature for HCC. Our study is expected to be helpful to predict or partly improve the prognosis of HCC patients.

## Methods

### Research objects

Mutation Annotation Format (MAF) files of 365 HCC patients were downloaded from The Cancer Genome Atlas (TCGA) database (https://tcga-data.nci.nih.gov/tcga/). Then we also downloaded the mRNA expression profile and the corresponding clinical information of 371 HCC patients from TCGA database, of which 365 patients had complete survival information (detailed clinical information was shown in Table 1). Moreover, other 237 HCC patients’ clinical information and mRNA data were obtained from Liver Cancer-RIKEN JP (LIRI-JP) dataset in International Cancer Genome Consortium (ICGC) database (https://icgc.org/).

**Table 1:** Clinicopathological characteristics of HCC sampless from TCGA database

Parameters	OS Status		х^2^	*P*-value
	Alive (N = 234)	Dead (N = 131)		
*Age* (Mean±SD)	58.02±13.69	61.79±13.68	0.11863	0.7305
*Gender*			3.2286	0.07236
Female	68	51		
Male	166	80		
*Pathologic stage*			23.022	3.996e-05
i	127	43		
ii	58	26		
iii	38	45		
iv	1	3		
Unknown	10	14		
*Race*			9.8764	0.05256
Asian	116	44		
White	101	77		
Black or african american	10	7		
American indian or alaska	2	0		
Unknown	5	3		

Additionally, another two datasets were downloaded from Gene Expression Omnibus (GEO) database (https://www.ncbi.nlm.nih.gov/geo/). GSE83148 comprised 6 normal liver tissue samples and 122 hepatitis B virus (HBV) infected liver tissue samples, totally 128 samples. GSE121248 contained 107 samples, including 37 HBV-positive HCC patients’ adjacent samples and 70 HBV-positive HCC patients’ tumor samples. The data in these two datasets were both detected on Affymetrix Human Genome U133 Plus 2.0 Array platform.

### LASSO Cox regression analysis

Based on gene expression, the HCC samples were subjected to univariate Cox regression analysis, after which the genes significantly related to the prognosis of HCC patients were screened with threshold *P* < 0.01. The optimal HCC prognostic related genes were further selected via LASSO Cox regression analysis using glmnet package of R [23]. Based on the optimal genes, all samples’ Risk score can be calculated via the following formula:

$$\ {\text{Risk}}\;{\text{Score}} = \sum\limits_{{{\text{i}} = 1}}^{{\text{n}}} {{\text{Coef}}_{{\text{i}}} } * {\text{X}}_{{\text{i}}}$$

Coefi was the risk coefficient calculated via LASSO Cox regression analysis, and Xi referred to mRNA expression here.

Then, according to the median of Risk score, all HCC samples were divided into high and low risk groups.

### Differential expression analysis

We utilized limma package [24] of R (version 3.5.2) to conduct differentially expressed gene (DEG) analysis. Significant DEGs were screened basing on |log_2_FC| >1 and FDR ≤ 0.05.

### Enrichment analysis

The functional enrichment analysis was then performed on these significant DEGs using “clusterProfiler” [25] of R, including Gene Ontology (GO) and Kyoto Encyclopedia of Genes and Genomes (KEGG) enrichment. *P* value < 0.05 (adjusted by Benjamini and Hochberg (BH) method) was adopted to screen significantly enriched GO terms and KEGG pathways.

### Survival analysis

The OS rates of various groups were estimated according to Kaplan-Meier method, utilizing survival and survminer packages of R. The significance of difference was determined by log rank test.

### Immune cell infiltration analysis

The relative proportions of various immune cells in every sample were calculated using software CIBERSORT [26]. Basing on gene expression matrix, relative proportions of infiltrating immune cells could be characterized according to the deconvolution algorithm. For each sample, the CIBERSORT output estimated proportions sum up to one.

### Nomogram building

Nomogram is an important tool to predict the prognosis of cancer patients. Thus, we utilized all independent prognostic factors obtained from multivariate Cox regression analysis to construct Nomogram, predicting 1, 3 and 5-years OS of HCC patients (using rms (Regression Modeling Strategies) package of R (https://CRAN.R-project.org/package=rms)). The calibration curve was drawn to test the prognostic performance of Nomogram.

### Drug target predictions

Genomics of Drug Sensitivity in Cancer (GDSC) database (https://www.cancerrxgene.org/) has been the largest public database including tumor cell drug sensitivity and tumor treatment genome data. Herein, this database was used to predict the corresponding medication information of genes, in order to explore the correlation between gene and drug sensitivity (ANOVA analysis, *P* value < 0.05).

### Statistical analyses

All independent prognostic indicators for HCC patients were determined by multivariate Cox regression analysis. Immune cell infiltration difference was tested by Wilcoxon signed rank sum test, and *p* < 0.05 was considered significant. All statistical analyses were conducted in R software v3.5.2.

## Results

### Identification of HBV-positive HCC related genes

**Fig. 1:**
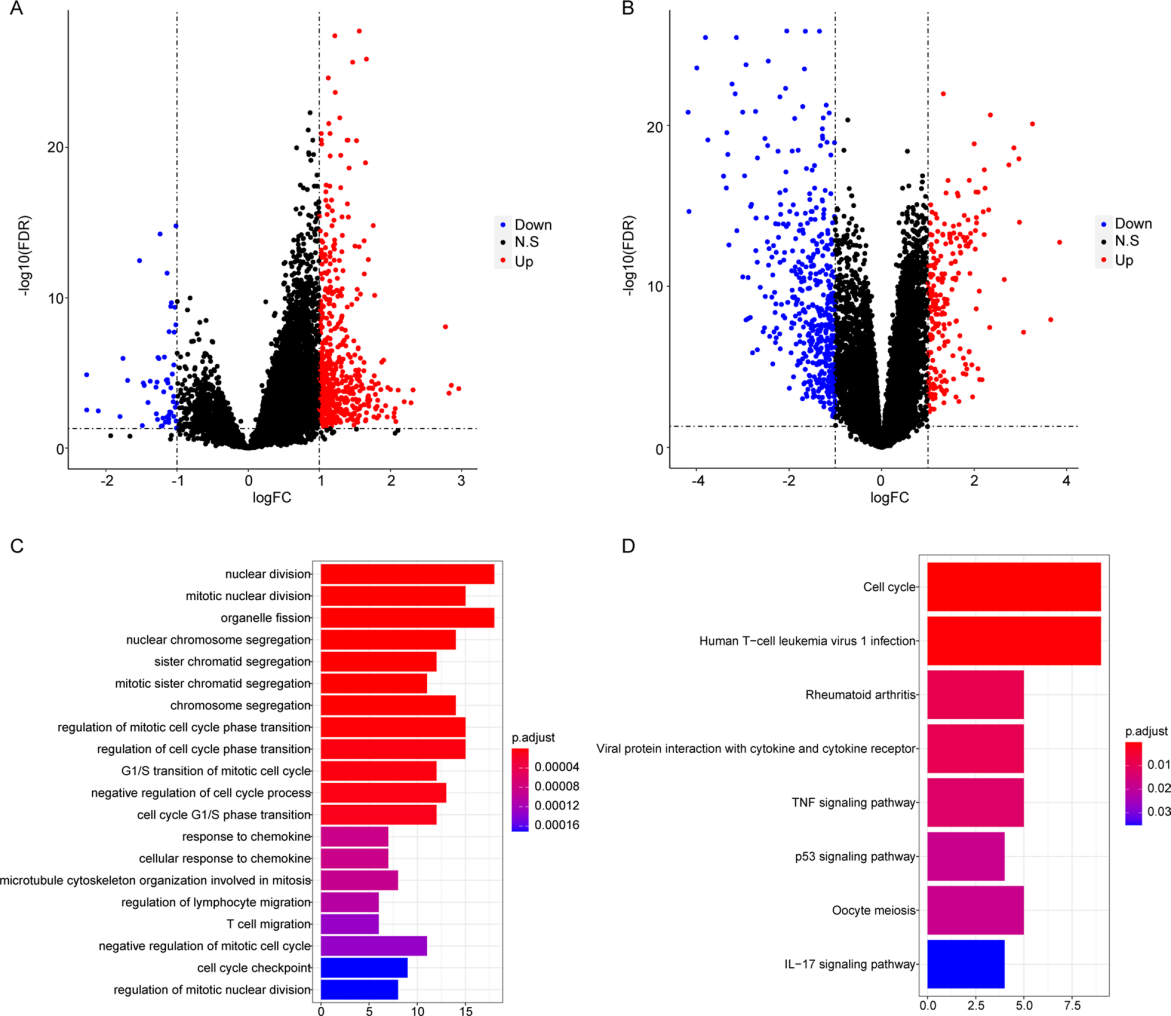
Identification of HBV-positive HCC related genes. **A**, **B** The identified DEGs in GSE83148 dataset and GSE121248 dataset, respectively. **C** The top 20 significantly enriched GO terms. *X*-axis: the number of enriched genes; *Y*-axis: names of GO terms. **D** Eight significantly enriched KEGG pathways. *X*-axis: the number of enriched genes; *Y*-axis: names of pathways

Firstly, basing on the data in GSE83148, we have conducted a differential expression analysis on normal liver tissue and HBV-positive liver tissue samples. Compared with normal liver samples, a total of 614 DEGs were identified in HBV-positive liver samples, comprising 561 upregulated genes and 53 downregulated genes (Fig. 1 A). Additionally, in GSE121248 dataset, compared with HBV-positive adjacent samples, there were 680 DEGs in HBV-positive HCC samples, including 227 upregulated genes and 453 downregulated genes (Fig. 1B).

We found that there were 106 overlapped DEGs between these two datasets (Additional file 1: Table S1), which were probably specific genes related to the development from HBV to HCC. In GSE83148, 94 overlapped genes were upregulated, and 12 genes were downregulated in HBV-positive samples. In GSE121248, there were 46 upregulated overlapping genes and 60 downregulated genes in HBV-positive HCC samples. Then the 106 DEGs were significantly enriched in 213 GO terms (top 20 terms, Fig. 1C) and 8 KEGG pathways (Fig. 1D). All detailed functional enrichment results were summarized in Additional file 2: Table S2.

### HCC patients with distinct prognosis could be divided based on HBV-positive HCC related genes

**Fig. 2:**
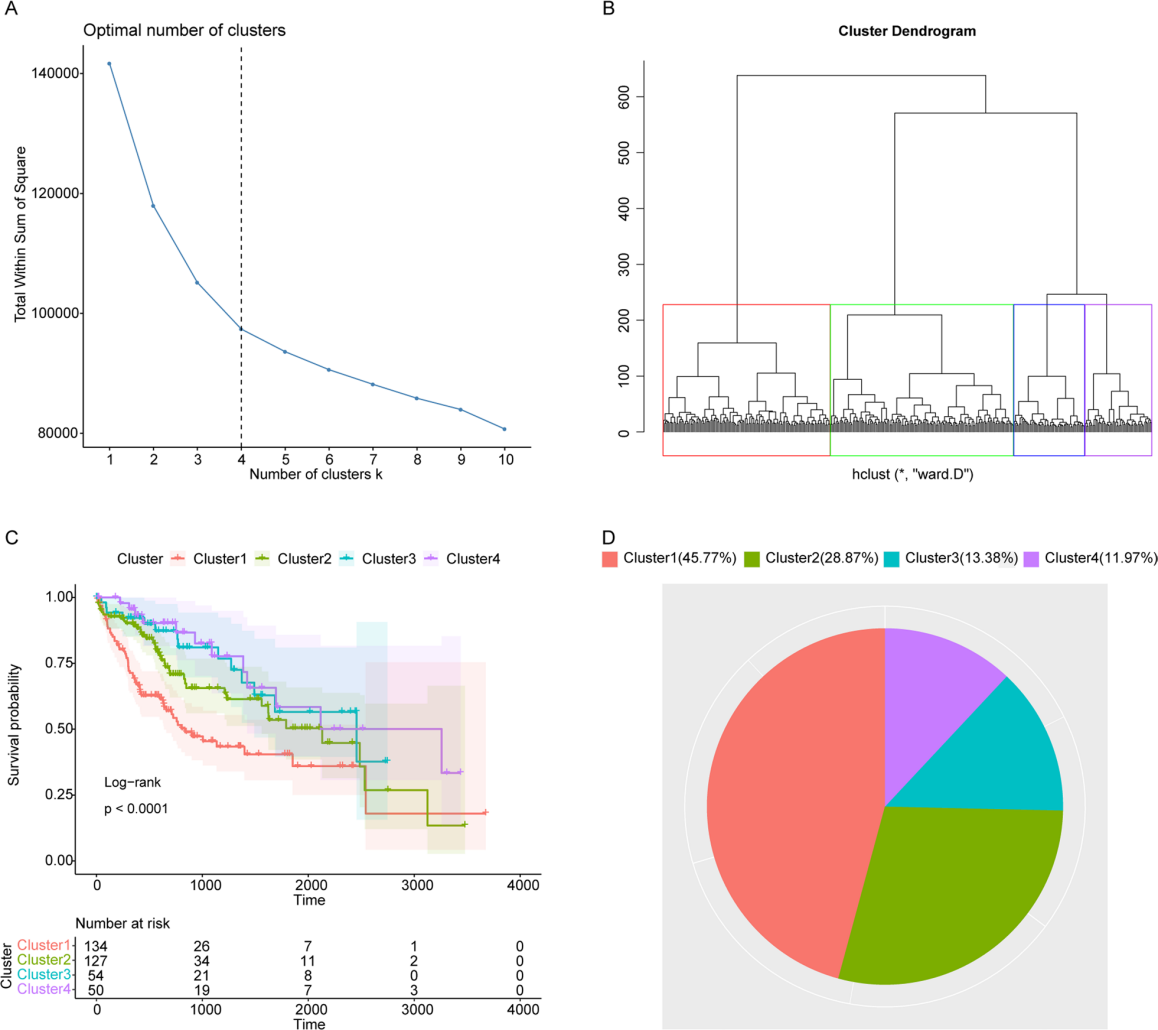
The cluster analysis results of the HCC samples. **A** Elbow diagram indicated that the optimal number of clusters was k = 4. **B** The cluster dendrogram of HCC samples. Different colors represent different clusters. **C** Kaplan Meier survival curve. The P value was calculated based on log-rank test. **D** The distribution of HBV-positive HCC patients in various clusters

Subsequently, basing on the expressions of the 106 HBV-positive HCC related genes, the 365 HCC samples in TCGA database were subjected to cluster analysis. The results of sum of the squared errors (SSE) indicated that the optimal clusters should be k = 4 (Fig. 2 A), then all samples were clustered into 4 categories (Fig. 2B). After conducting Kaplan-Meier (KM) survival analysis, we found significantly differential OS among 4 clusters’ HCC patients. HCC patients in Cluster1 and Cluster2 had worse prognosis, while patients in Cluster3 and Cluster4 had better prognosis (Fig. 2C). After checking the clinical information of these 365 HCC patients, we found that 142 HCC patients were HBV-positive, and the rest 223 patients had unclear HBV information. Regarding the 142 HBV-positive patients, among which, 65 (45.8%) and 41 (28.9%) patients were clustered in poor prognostic Cluster1 and Cluster2, respectively (Fig. 2D). Whereas, only 19 (13.4%) and 17 (11.9%) patients were clustered into good prognostic Cluster3 and Cluster4, respectively (Fig. 2D).

### The risk score based on 11 genes could reliably predict the prognosis of HCC patients

**Fig. 3:**
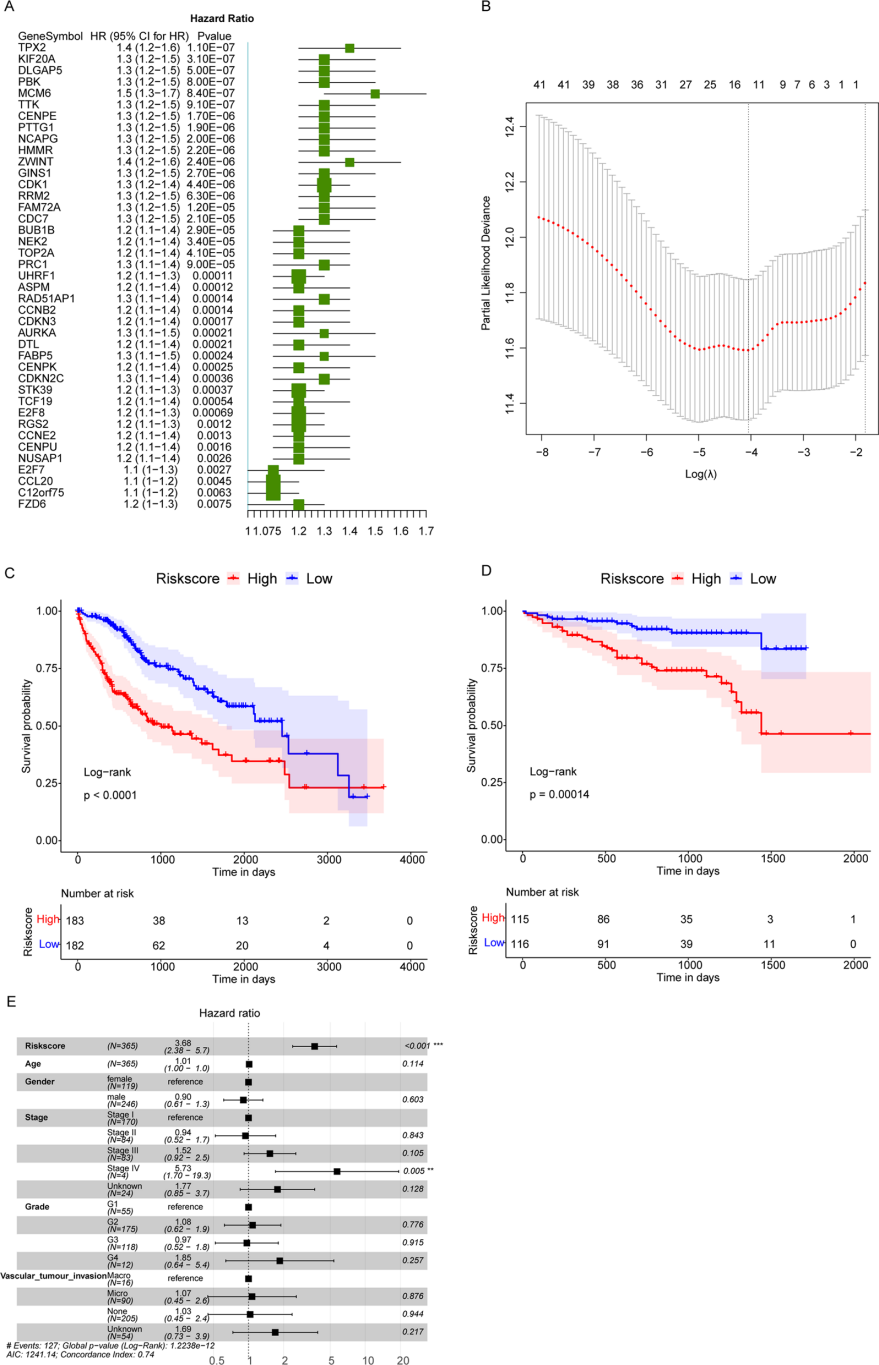
The construction of predictive Risk score for HCC. **A** HCC prognostic related genes. HR: Hazard ratio, 95% CI 95% confidence interval. **B** The optimal gene number was 11, corresponding to the lowest lambda. *X*-axis: log(lambda); *Y*-axis: partial likelihood deviance. **C**, **D** Kaplan Meier survival curve of HCC samples in TCGA and ICGC databases, respectively. *P* value was based on log-rank test. **E** Multivariate Cox regression analysis results. HR > 1 means higher death risk, while HR < 1 is contrary

All HCC samples in TCGA database was then subjected to an univariate Cox regression analysis taking expression values of 106 HBV-positive HCC related genes as continuous variable, and the Hazard ratio (HR) of each gene was calculated. HR < 1 represented the beneficial role of gene in patient prognosis, while HR > 1 meant higher risk for patient poor prognosis. Then we obtained a total of 42 significant genes (P value < 0.01), all of which were risk genes with HR > 1 (Fig. 3 A). LASSO Cox regression analysis was subsequently performed on these 42 selected genes. According to the lowest lambda value, the corresponding optimal number of gene was 11 (Fig. 3B). The optimal genes included HMMR (hyaluronan mediated motility receptor), MCM6 (minichromosome maintenance complex component 6), TPX2 (TPX2 microtubule nucleation factor), KIF20A (kinesin family member 20 A), CCL20 (C-C motif chemokine ligand 20), RGS2 (regulator of G protein signaling 2), NUSAP1 (nucleolar and spindle associated protein 1), FABP5 (fatty acid binding protein 5), FZD6 (frizzled class receptor 6), PBK (PDZ binding kinase), and STK39 (serine/threonine kinase 39).

Then gene expression was weighted with regression coefficient of LASSO Cox regression analysis to establish a predictive prognostic Risk score model, Risk score = (HMMR*0.142113311)+(MCM6*0.084558199)+(TPX2*0.285390199)+(KIF20A*0.083398740) )+(CCL20*0.029641894)+(RGS2*0.031218559)+(NUSAP1*-0.449566369)+(FABP5*0.002614688)+(FZD6*0.017389305)+(PBK*0.094068871)+(STK39*0.032363425). Thus, the Risk score could be calculated for each sample. All HCC samples, in TCGA database (training set) and ICGC database (validation set), were divided into high and low risk groups, basing on the median of Risk score. We found that in both data sets, HCC patients with high Risk score had poorer OS compared with low Risk score patients (Fig. 3C, D). Moreover, a multivariate Cox regression analysis was then conducted on age, gender, Stage, Grade, Vascular tumour invasion and Risk score in order to find independent prognostic indicators for HCC patients (Fig. 3E). Our results showed that Risk score and Stage were significantly related to OS of HCC patients. Those HCC patients with higher Risk score had poorer OS compared with lower Risk score patients (HR = 3.68, 95%CI 2.38–5.7, *P* < 0.001). Collectively, the Risk score built based on HMMR, MCM6, TPX2, KIF20A, CCL20, RGS2, NUSAP1, FABP5, FZD6, PBK and STK39, could well predict the prognosis of HCC patients.

### Nomogram had good prognostic prediction performance

**Fig. 4:**
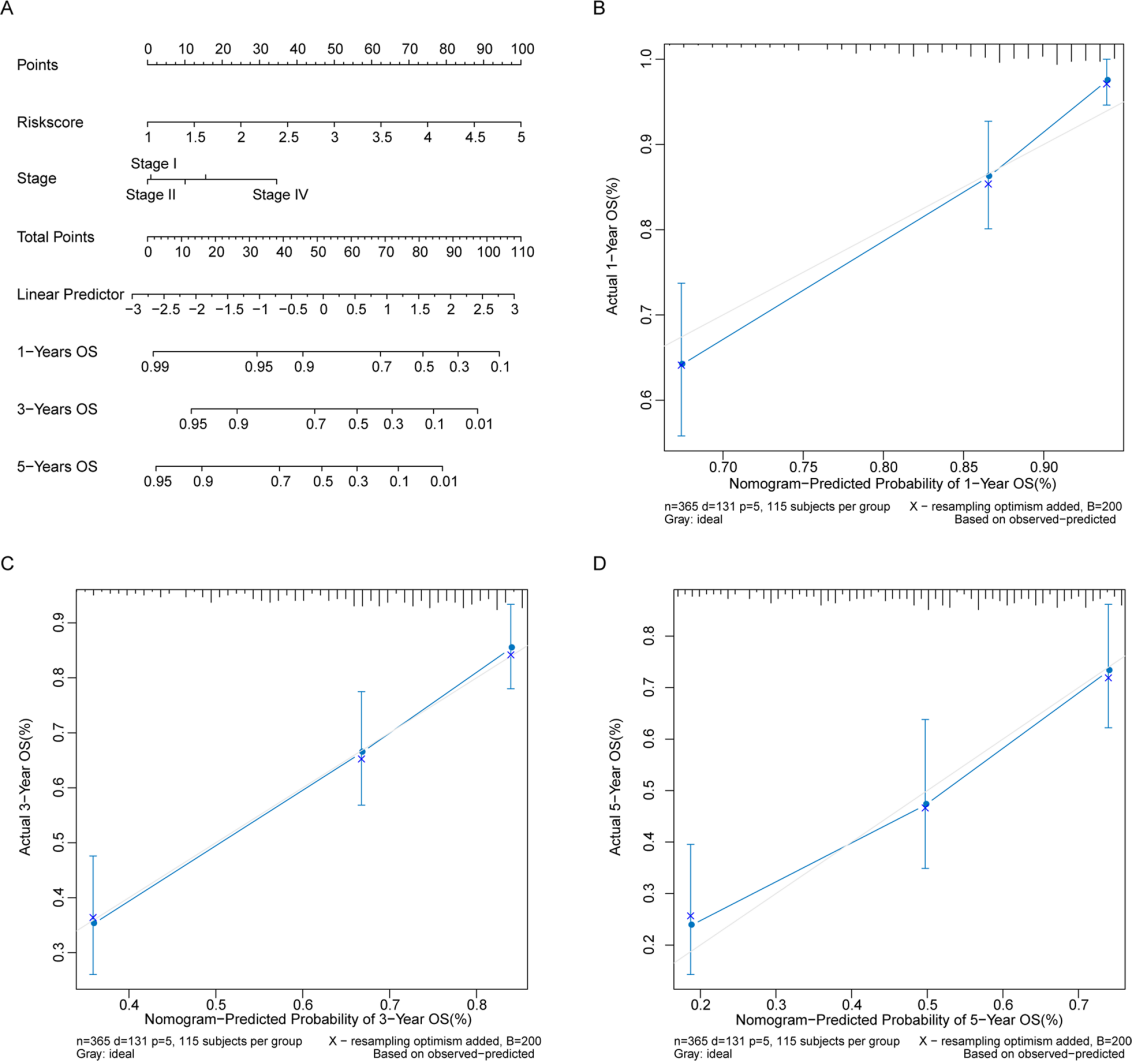
Nomogram could predict the OS of HCC patients. **A** Nomogram could predict 1-year, 3-year, and 5-year OS of HCC patients. **B**–**D** Nomogram calibration curves of 1-year, 3-year, and 5-year, respectively. *X*-axis: predicted survival probability; *Y*-axis: actual survival probability

Nomogram was then built based on the two independent prognostic factors, comprising Stage and Risk score (Fig. 4A). For each HCC patient, three upward lines would determine the Points got from the Nomogram, the sum of the points was located on the Total Points axis. A line downward from Total Points axis finally determined the 1, 3 and 5-years OS of HCC patients. The 1 and 3-years calibration curves were well matched the ideal curve (the line passing through origin with a slope of 1), which implied that the Nomogram had a relatively good prognostic predictive effect (Fig. 4B and D).

### The differential immune cell infiltration and differential mutated genes between high and low risk HCC patients

**Fig. 5:**
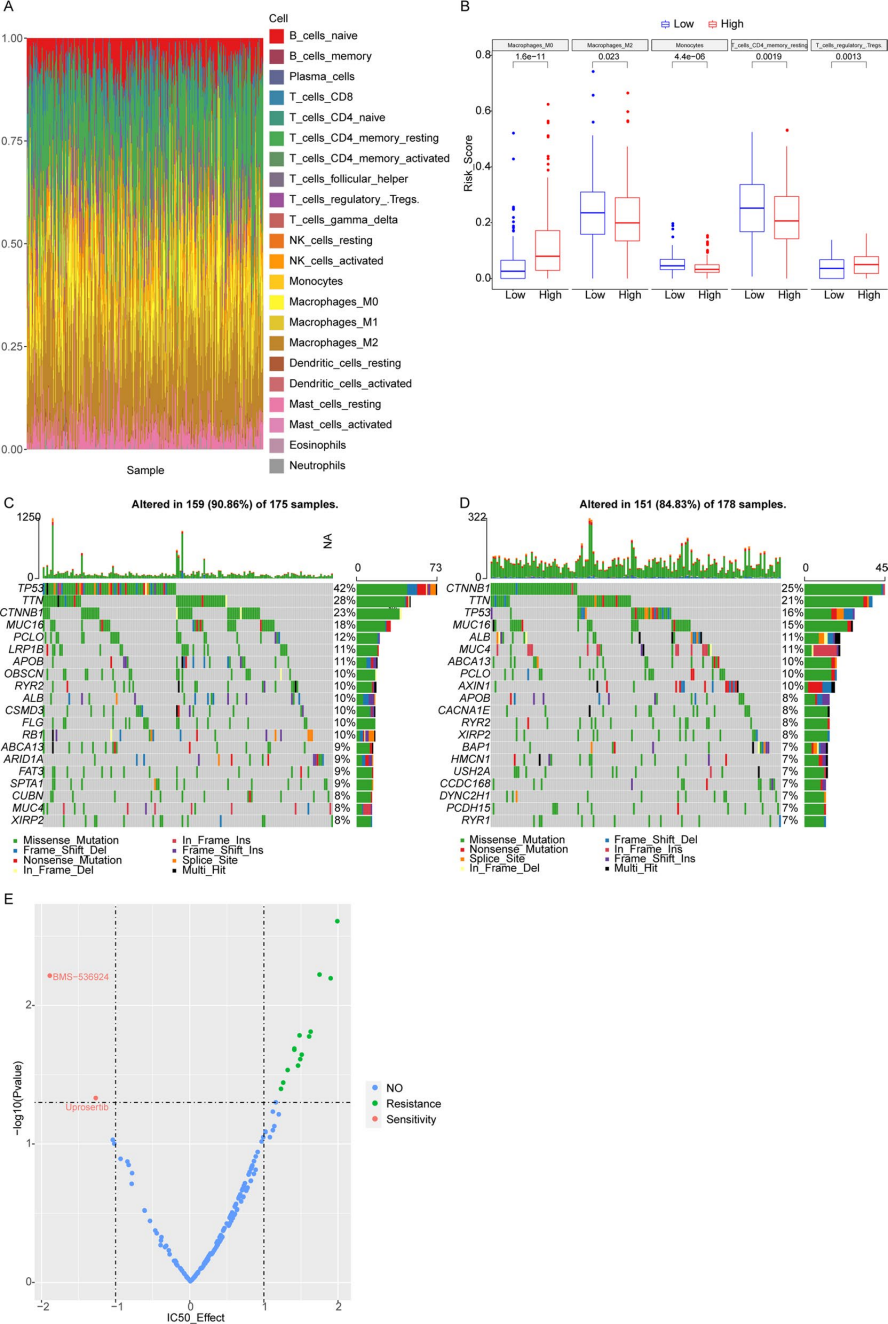
Immune cell infiltration difference between high and low risk HCC patients. **A** Immune cells’ infiltration of 365 HCC samples in TCGA database. **B** 5 types of significantly differentially infiltrated immune cells between high and low risk HCC patients. **C**, **D** The top 20 genes with highest mutation rates in high and low Risk score HCC patients, respectively. **E** Drug sensitivity results. *X*-axis: IC50 score

Combining LM22 feature matrix with CIBERSORT method, various immune cells’ infiltration was estimated in high and low risk HCC patients. The detailed immune cells’ infiltration of 365 HCC samples in TCGA database has been summarized in Fig. 5 A, which implied that tumor immune cell infiltrating heterogeneity of different individuals. Between high and low Risk HCC patients, totally 5 types of immune cells, including Macrophages M0, Macrophages M2, Monocytes, T cells CD4 memory resting, and T cells regulatory Tregs, were significantly differentially infiltrated (Fig. 5B). We found that in high risk HCC patients, TP53 (tumor protein p53) showed the highest mutation rate (42%) (Fig. 5 C), while in low risk patients, CTNNB1’s (catenin beta 1) mutation rate 25% was highest (Fig. 5D). Meanwhile, the ratio of HBV-positive HCC patients (46.4%) in high Risk score patients was higher than that in low Risk score patients (31.3%). We have searched the medication information targeting TP53 mutation in GDSC database, which indicated that Uprosertib and BMS-536,924 had high sensitive to TP53 mutated HCC patients (Fig. 5E).

## Disscussion

Despite great efforts have been devoted to improve the outcome of HCC patients, little effect has been brought to prolong the OS of HCC patients [27]. In this study, we have integrated the HCC related data in TCGA, ICGC, and GEO databases, and identified 106 genes which were probably specific genes related to the development from HBV to HCC. Furthermore, we built a relatively reliable predictive Risk score for HCC based on 11 genes and high Risk score was an unfavorable prognostic factor for HCC.

In HCC, HBV infection and high virus load has been widely considered the risk factors [28]. Whereas, the potential influence of HBV infection on the progression or prognosis of HCC largely remains unclear. Therefore, firstly, we have identified the possible HBV-positive HCC related specific genes. Basing on the data in GEO database, we have identified 614 DEGs and 680 DEGs in normal liver samples vs. HBV-positive liver samples and HBV-positive adjacent samples vs. HBV-positive HCC samples, respectively. Among that, 106 overlapped DEGs were probably HBV-positive HCC related specific genes, which were significantly enriched in 213 GO terms and 8 KEGG pathways. Some of these KEGG pathways have been reported in HCC previously. For example, cell cycle pathway, it has been suggested that HBV might deregulate cell cycle control to form a cellular environment conducive to infection, thereby inducing the malignant transformation of infected hepatocytes [29]. Moreover, HCC cell growth might be inhibited by inducing cell cycle arrest and apoptosis [30]. Moreover, several well known tumor related pathways were also observed, such as TNF signaling pathway, p53 signaling pathway, IL-17 signaling pathway, and so on. Among them, some genes/ lncRNAs/ proteins were evidenced to involve in the regulation of proliferation or metastasis of HCC via TNF signaling pathway [31–33]. A recent report has demonstrated the potential important role of p53 signaling pathway in the development of HBV-related HCC [34], which was also support our notion indirectly. On the other hand, based on these 106 DEGs, all HCC samples in TCGA could be divided in 4 clusters with different prognosis, and most HBV-positive HCC patients (74.6%) had worse prognosis. Our findings implied the importance of these 106 HBV-positive HCC related specific genes.

Subsequently, univariate Cox and LASSO Cox regression analysis were conducted on these 106 genes and the HCC data in TCGA, then 11 optimal genes were selected to build a Risk score, including HMMR, MCM6, TPX2, KIF20A, CCL20, RGS2, NUSAP1, FABP5, FZD6, PBK, and STK39. High risk HCC patients were evidenced to have worse OS in both training set and validation set. Moreover, Risk score was an independent prognostic factor for HCC. We also found some clues of the optimal genes to indirectly support our prognostic model. HMMR was found to be dysregulated in HBV related HCC [35], besides HMMR was also identified as a candidate gene involving the mechanisms behind HCC in China [36]. Whereas, the prognostic value or exact role HMMR has been seldom studied in HBV related HCC, which still needs to be clarified then. MCM6 has been suggested as a potential prognostic biomarker for HCC [37]. Moreover, MCM6 was also found to play a vital role in the progression of HCC in Chinese Zhuang population [38]. These studies were both in line with our data. TPX2 has been associated with the carcinogenesis and proliferation of HBV-related HCC [39, 40], while more details are not clear. KIF20A was reported to be related to the OS of HCC patients [41], besides, a prognostic marker based on 12 genes included KIF20A showed good predictive effect [42], both of which supported our results. High expression of CCL20 has been documented to be correlated with the poor prognosis of HCC patients [43]. Moreover, NUSAP1 [44], FABP5 [45], PBK [46], and STK39 [47] have been indicated to associate with the progression, metastasis, invasion, or prognosis of HCC directly or indirectly, which provided more evidence of our Risk score. Despite few studies of RGS2 and FZD6 were found in HCC, which deserved more exploration in the future. All above data evidenced that our Risk score was a relatively reliable prognostic predictive tool for HCC. Additionally, our Nomogram based on Risk score and stage had a good performance, which might make our Risk score more convincing.

## Conclusion

In conclusion, via our joint analyses preformed on the HCC related data downloaded from three public databases, we have firstly revealed a prognostic signature based on HBV related specific genes in HCC. The Risk score constructed basing on 11 genes has a good prognostic predictive performance, and high Risk score is a poor prognostic indicator.

## Supplementary Information

**Additional file 1**. **Table S1** The 106 overlapped DEGs.

**Additional file 2**. **Table S2** The detailed functional enrichment results.

## Data Availability

The datasets generated and analysed during the current study are available in The Cancer Genome Atlas (TCGA, https://tcga-data.nci.nih.gov/tcga/), International Cancer Genome Consortium (ICGC, https://icgc.org/), and Gene Expression Omnibus (GEO, https://www.ncbi.nlm.nih.gov/geo/, accession number: GSE83148 and GSE121248).

## Abbreviations

HCC: Hepatocellular carcinoma
HBV: Hepatitis B virus
HCV: Hepatitis C virus
MAF: Mutation annotation format
TCGA: The cancer genome Atlas
ICGC: International cancer genome consortium
GEO: Gene expression omnibus
DEGs: Differentially expressed genes
GO: Gene ontology
KEGG: Kyoto encyclopedia of genes and genomes
OS: Overall survival
